# Evaluation of Serum Pentraxin-3 Level in Patients with Acute Myocardial Infarction Compared with Control Group

**DOI:** 10.30699/IJP.2021.116962.2268

**Published:** 2021-05-09

**Authors:** Alireza Firouzjahi, Saeedeh Eris, Seyed Farzad Jalali, Ali Bijani, Mohammad Ranaee

**Affiliations:** 1 *The Clinical Research Development Unit of Rouhani Hospital, Babol University of Medical Sciences, Babol, Iran *; 2 *Cellular and Molecular Biology Research Center, Health Research Institute, Babol University of Medical Sciences, Babol, Iran*; 3 *Student Committee Research, Babol University of Medical Sciences, Babol, Iran*; 4 *Social Determinants of Health Research Center, Health Research Institute, Babol University of Medical Sciences, Babol, Iran*; 5 *Cancer Research Center, Health Research Institute, Babol University of Medical Sciences, Babol, Iran*

**Keywords:** Pentraxin 3, Troponin I, Myocardial ischemia, Coronary angiography

## Abstract

**Background & Objective:**

The aim of this study was to measure serum pentraxin 3 (PTX3) in patients with acute myocardial infarction (MI) and compare it with the control group.

**Methods:**

In this case-control study, 60 patients with MI (±ST-segment elevation) were included in the case group , and those with symptoms suspicious for coronary artery disease (CAD) and with no abnormal findings in angiography and troponin I level less than 99th percentile of normal population were included as a control group (*N*=30). Serum PTX3 and troponin I were measured.

**Results:**

In this study, 60 patients including 34 men and 26 women were included in the case group (mean age: 61.4±8.86 years in non–ST-segment elevation myocardial infarction [NSTEMI] subgroup and mean age: 57.9±9.49 years in ST-segment elevation myocardial infarction [STEMI] subgroup), as well as 13 men and 17 women as the control group (mean age: 55.47±10.09 years). PTX3 level was higher in MI cases (1128.4±1205 pg/mL) compared to controls (394.5±170.40 pg/mL) (*P*=0.001). There was no relationship between ejection fraction (EF) and PTX3 level in the MI group. The area under the ROC curve (AUC) of PTX3 in MI was presented by 0.828 (AUC=0.828) (*P*>0.001). We defined three different cutoffs for PTX in this study, in which the cutoff ≥400 pg/mL had the highest sensitivity (92%), and the cutoff ≥700 pg/mL had the highest specificity (97%).

**Conclusion:**

According to the results of this study, PTX3 as an inflammatory marker showed higher level in patients with MI, especially in STEMI cases. Therefore, combined evaluation of troponin I and PTX3 levels would be associated with more accuracy in diagnosis of MI.

## Introduction

Cardiovascular diseases (CVDs) are among the most common causes of mortality and morbidity all over the world. Previous studies have suggested that different factors (such as age, gender, diabetes mellitus, hyperlipidemia, and hypertension) are responsible for CVD etiology (1); however, some pieces of evidence showed that inflammation makes atherosclerotic lesions prone to the erosion and rupture (1).

Acute coronary syndrome (ACS) includes unstable angina, myocardial infarction (MI) without ST-segment elevation (non-Q wave MI or non–ST-segment elevation myocardial infarction [NSTEMI]), and MI with ST-segment elevation (Q wave elevation or ST-segment elevation myocardial infarction [STEMI]) (2).

Clinical differentiation between cardiac and non-cardiac pains needs a combination of the patient’s history, electrocardiogram (ECG), and biomarkers (2).

In the pentraxin (PTX) family, in addition to C-reactive protein (CRP), PTX3 (defined as a long PTX) is locally secreted by smooth muscle and endothelial cells or by monocytes/macrophages exposed to the first inflammatory signals (such as interleukin 1, tumor necrosis factor-α, oxidized low-density lipoprotein, and bacterial products) (3). PTX3 also increases in ACS and congestive heart failure (CHF), which shows its effective role in local vascular inflammation and cardiovascular system damage (4-6).

The peak level of PTX3 will be reached seven hours after presenting symptoms in STEMI and will have an independent predictive role in three-month mortality when measured during the first day (4).

 This study was designed to measure PTX3 in patients with MI and compare it with the control group.

## Material and Methods

This study was approved by the Local Ethics Committee of Babol University of Medical Sciences, and informed consent has been obtained from all individuals included in this study. 

In this case-control study, a total of 60 patients (34 men and 26 women) with coronary artery disease (CAD) were enrolled, who were referred to Ayatollah Rohani Hospital; Babol, Iran, also, 30 control subjects were included in the control group. All of these individuals (*N*=90) were categorized into three groups as follows:

1. MI with STEMI in ECG and rise in cardiac enzymes 

2. MI without NSTEMI in ECG and rise in cardiac enzymes

3. Control group: Sex and age-matched (40-70 years) patients were selected based on symptoms suspicious for CAD and no abnormal findings in angiography and troponin I level less than 99th percentile of normal population.

Subjects with CHF, renal and liver failures, and acute inflammatory diseases, as well as patients receiving steroids, immunosuppressant drugs, and non-steroidal anti-inflammatory drugs (NSAIDs), except for low-dose aspirin, were excluded from the study.

During the first 24 hours (preferably during the first 12 hours) of patients’ admission, 5 cc venous blood was taken, and PTX3 and troponin I were measured using the Human Pentraxin 3 Kit (Biovendor Company, Brno, Czechia) by the enzyme-linked immunosorbent assay (ELISA) method. Angiography was done based on clinical status and along with diagnosis and treatment process during the first to third day of admission, except in those with critical status that was done urgently.

Demographic data, such as age, gender, weight and height, hypertension, hypercholesterolemia, family history of CVD, body mass index (BMI), and smoking, were recorded in a checklist (7). MI was classified as follows (8):

1. Anterior: septal (v1-v2) - mid anterior (v3-v4) - lateral (v5-v6)

2. Extensive anterior (v1-v6)

3. Inferior (II, III, and aVF)

4. Right ventricle (Rv3-Rv4)

5. Posterior (tall R wave and ST depression in v1-v2)

Data were analyzed using Chi-square, *t*, ANOVA, and Mann-Whitney tests.

## Results

In this study, 30 patients with STEMI, 30 with NSTEMI, and 30 controls were compared. There was no significant difference between all groups regarding age, sex, cigar, and other demographic variables, except in the history of hyperlipidemia (see Table 1).

No history of ischemic heart disease (IHD) was reported in the control group, but there were 21 cases (35%) with IHD in the MI group (*P*>0.001).

PTX3 level was significantly higher in the cases with MI (1128.4±1205 pg/mL) compared to the control group (394.5±170.40 pg/mL; *P*>0.001).

**Fig. 1:**
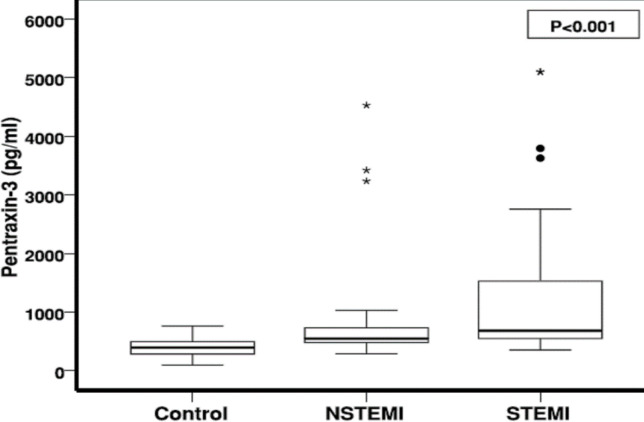
Comparing the mean PTX-3 level between MI and normal group

Kruskal-Wallis and Mann-Whitney tests showed a significant difference between PTX3 level in the normal group to each of the STEMIs (*P*>0.001) and NSTEMIs (*P*=0.053). As shown in Figure 1, PTX3 level was significantly higher in the STEMI group (1367±1358 pg/mL) than in the NSTEMI (890±996 pg/mL) group.

Troponin level was also significantly higher in the STEMI group (11.96±6.4 ng/mL) than in the NSTEMI (5.62±6.3 ng/mL) group. 

Spearman correlation coefficient was 0.338 for troponin and PTX3 (*P*=0.134) and 0.201 for age and PTX3 (*P*=0.375). 

No one in the MI group had ejection fraction (EF) upper than 55%. In the STEMI group, 20 cases (66.7%) and in the NSTEMI group, 14 cases (46.7%) had EF=30%-45%. There was no significant difference between these two groups.

There was no relationship between EF and PTX3 in the MI group. However, there were higher PTX and troponin levels in lower EF (not significant). Forty-one cases had EF<45% with PTX 1298±1335, and 19 cases had EF≥45% with PTX 761±766 (*P*=0.076).

Anterior MI was seen in 19 (63.3%) NSTEMI and 17 (56.2%) STEMI cases. Inferior MI was reported in seven NSTEMI and 11 STEMI patients.

Left anterior descending coronary artery (LAD) was the most-seen involved vessel in 38 (63.3%) cases of both MI groups, and then right coronary artery (RCA) had the second position in 16 (26.6%) cases. 

Involved vessels in angiography showed a significant relationship with the serum PTX3 level; it was higher in LAD, then RCA, and left circumflex coronary artery (LCX) (*P*>0.001). Five cases had extensive anterior MI with PTX3 level 2465±2170, and 55 cases had other MI with PTX 1007±1028 (*P*=0.042). 

There was a significant relationship between the numbers of involved vessels and PTX (*r*=0.516, *P*>0.001).

**Fig. 2:**
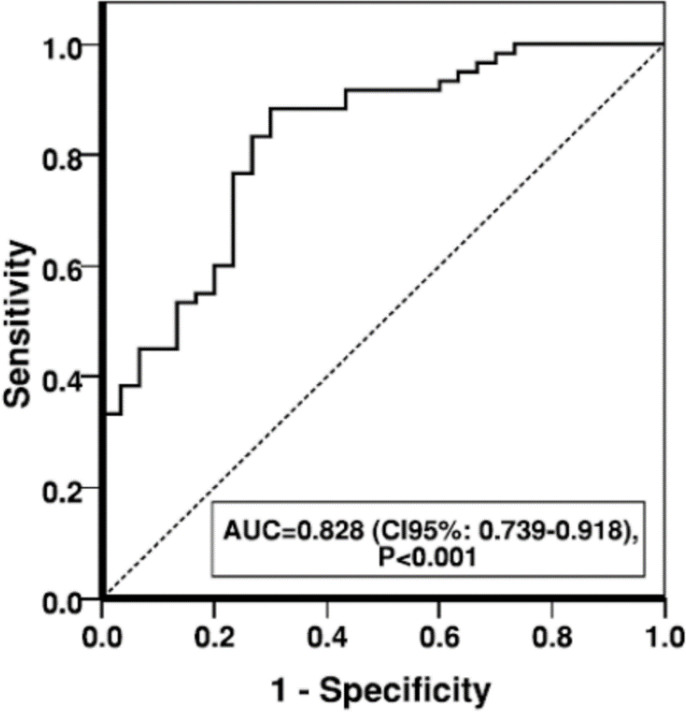
Area under curve of PTX-3 serum level in MI patients

As shown in Figure 2, sensitivity and specificity of PTX in MI was presented by the area under the ROC curve (AUC) of 0.828, showing a high overall discriminatory ability of PTX in MI (AUC=0.828; 95% CI, 0.739-0.918; *P*>0.001). 

In patients suspicious for CAD without any abnormal findings in angiography and troponin I level less than 99th percentile of normal population, there was no significant relationship between the PTX serum level and demographic variables.

Three different cutoffs were offered for PTX and for each cutoff, sensitivity, specificity, positive predictive value & negative predictive value have been calculated. As shown in Table 2, the cutoff ≥400 had the highest sensitivity, and the cutoff >700 had the highest specificity; however, the best sensitivity and specificity concurrently is ≥500 pg/mL.

**Table 1:** Comparing demographic variables between controls and MI cases

P-value	Group		Variables	
	MI	Control		
**0.23**	34 (56.7)	13 (43.3)	male	**Gender**
	26 (43.3)	17 (56.7)	Female	
**0.13**	41 (68.3)	25 (83.3)	No	**Smoking **
	19 (31.7)	5 (16.7)	Yes	
**0.006**	40 (66.7)	28 (93.3)	No	**History of hyperlipidemia**
	20 (33.3)	2 (9.1)	Yes	
**0.75**	40 (66.7)	21 (70)	No	**History of diabetes mellitus**
	20 (33.3)	9 (30)	Yes	
**0.13**	38 (63.3)	14 (46.7)	No	**History of hypertension**
	22 (36.7)	16 (53.3)	Yes	
**0.72**	46 (76.7)	24 (80)	No	**Family history of heart diseases**
	14 (23.3)	6 (20)	Yes	
**0.053**	59.65 (9.3)	55.47(10.1)	**Age (Mean; SD); years**	

**Table 2:** Sensitivity, specificity, positive predictive value, and negative predictive values of 3 different cutoffs for PTX in MI patients

**PPV** **(CI95%)**	**NPV** **(CI95%)**	**Positive likelihood ratio** **(CI95%)**	**Negative likelihood ratio** **(CI95%)**	**Specificity** **(CI95%)**	**Sensitivity** (CI*95%)	**Serum PTX level**
**79%** **(69-88)**	75% (56-94)	1.83 (1.27-2.64)	0.17 (0.07-0.42)	50% (32-68)	92% (85-99)	**≥** ** 400 pg/mL**
**87%** **(78-96)**	62% (47-78)	3.30 (1.70-6.41)	0.3 (0.18-0.49)	77% (62-92)	77% (66-88)	**≥ 500 pg/mL**
**96%** **(88-100)**	44% (32-56)	11.5 (1.63-81.12)	0.64 (0.52-0.79)	97% (90-100)	38% (25-51)	**≥** ** 700 pg/mL**

*Confidence interval

## Discussion

In this study, in MI and normal patients (who underwent angiography), there was no significant relationship between PTX and demographic variables. However, some studies have reported it as significant (3, 9).

Present results showed that in MI cases (STEMI and NSTEMI), PTX was significantly higher than the control group. The mean PTX level was also higher in the STEMI group compared to the NSTEMI group. This was also seen in previous studies. Rashtizadeh *et al*. in Iran (9), Ma *et al*. in China (10), and Eggers *et al*. in Canada (3) reported a significantly higher level of PTX in MI cases, although the studied populations and sample sizes were different compared to this study; however, a higher level of PTX as an inflammatory marker in ACS was commonly seen. 

In the present study, the numbers and locations of involved vessels and sites of MI had a significant relationship with PTX. PTX3 was significantly higher in LAD stenosis, extensive anterior MI, and higher numbers of involved vessels. This was not reported in other studies.

Rashtchizadeh *et al*. (9) reported a relationship between PTX level and age and hypertension that was not seen in the present study. In Eggers *et al.’s* study, PTX was independently related to female gender and cardiac troponin level in NSTE-ACS, but not to age or other cardiovascular factors (3).

In this study, troponin level was significantly higher in STEMI than in NSTEMI. In the MI group, there was no significant relationship between EF and PTX or troponin, but PTX3 was higher in lower EF, although it was clinically important and shows a higher inflammation level in MI cases. The sensitivity and specificity of PTX was high in MI patients.

Rashtchizadeh *et al*.'s study reported an AUC of 0.078 for PTX which had better diagnostic ability compared to high-sensitivity-CRP (hs-CRP) (9). In Rashtchizdeh *et al.’s* study, the studied population was diabetic patients that have strong risk factors for CVDs. They also compared the sensitivity and specificity of PTX and hs-CRP (9), which was not performed in this study. 

Kume *et al*. reported AUC=0.901 for PTX in ACS and concluded that the combination of troponin and PTX would diagnose the MI with higher accuracy (11).

In this study, among three cutoffs defined for PTX, it was seen that the cutoff level ≥400 pg/mL had the highest sensitivity, and the cutoff>700 pg/mL had the highest specificity. These cutoffs were defined in the present study because this kit is not used as a routine test in our country, and it is used for research. Thus, it should be evaluated more carefully in further studies.

The limitation of this study is that we could not evaluate in-hospital mortality (short-term follow-up) rate, which was considered as one of our goals. Thus, we were looking for a prognostic factor. However, due to the small statistical population (two cases of mortality), no statistical analysis was possible. We suggest that another study be done with more samples. 

## Conclusion

According to the results of this study, PTX3 was higher in patients with MI, especially in STEMI cases. Therefore, the combination of troponin I and PTX3 levels would diagnose MI with higher accuracy.

## Conflict of Interest

The authors declare no conflict of interest.
